# The research contribution of the Schistosomiasis Collection at the Natural History Museum (SCAN): highlights, challenges and future directions

**DOI:** 10.1186/s40249-025-01302-y

**Published:** 2025-04-18

**Authors:** Aidan M. Emery, Muriel Rabone, Toby Landeryou, Fiona Allan, David Rollinson

**Affiliations:** 1 Natural History Museum, Cromwell Rd, London, SW7 5BD UK; 2 Centre for Epidemiology and Planetary Health, School of Veterinary Medicine, Scotland’s Rural College, Inverness, IV2 5NA UK; 3 Global Schistosomiasis Alliance, Ealing Cross, 85 Uxbridge Road, Ealing, London, W5 5BW UK

**Keywords:** Schistosomiasis, Collections, Repositories, Genetic/genomic resources, Neglected tropical diseases

## Abstract

**Background:**

The Schistosomiasis Collection at the Natural History Museum (SCAN) is a repository of schistosomiasis-related specimens, the development of which was funded by the Wellcome Trust between 2011 and 2021. With a view to facilitating research by improving access to genetically diverse material, SCAN was built from legacy research collections of schistosomiasis-related specimens amassed over decades, with more recent collections made through partnership with large field-based projects.

**Methods:**

We identified the literature associated with SCAN from 2012 until 2024, using both database searches (search terms: SCAN, the schistosomiasis collection at the NHM and schistosomiasis) and citations of the publication which originally laid out the scope of the SCAN Collection. Studies were included if the SCAN publication was cited, and/or if the SCAN Collection was utilised in the work. Data extracted included year of publication, authors, whether and how SCAN was used in the work, and type of specimens used.

**Results:**

The literature includes 88 published works, demonstrating the utility of large field-based collections in supporting research. The collection comprises around half a million larval schistosomes originating from the field, with approximately 3000 specimen lots of lab-passaged adult parasites stored in liquid nitrogen. The Collection includes 11 schistosome species, the majority being the human pathogens *Schistosoma haematobium* and *S. mansoni*, while also including many livestock-associated species. Genome analysis of *S. haematobium* and S*. guineensis* samples indicate historical introgression or ongoing hybridisation. In order of representation, the collection includes *S. haematobium* (> 19,000 larval forms and eggs, and 550 specimen lots of laboratory passaged adult worms), *S. mansoni*, *S. japonicum*, *S. bovis*, *S. curassoni*, *S. mattheei*, *S. rodhaini* and *S. guineensis*, with *S. intercalatum*, *S. margrebowiei* and *S. spindale* represented only by laboratory-passaged isolates in liquid nitrogen. SCAN also includes around 210,000 snails, with the collection as a whole encompassing 27 countries.

**Conclusions:**

Improvements in DNA sequencing techniques have allowed genome-level data to be accessed from archived larval schistosomes and allowed retrospective analysis of samples collected decades ago. SCAN has been of use in exploring schistosome diversity, particularly with reference to hybridisation and drug resistance. Multiple author nationalities demonstrate the collaborative nature of research using the Collection, although more may need to be done in future, both to promote work led by developing countries and to ensure effective collaboration and sample sharing.

**Graphical Abstract:**

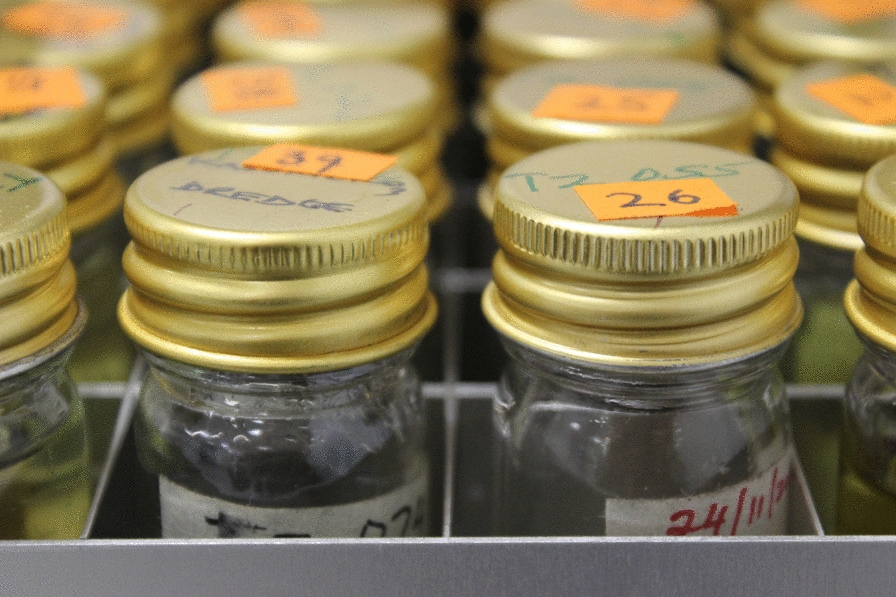

**Supplementary Information:**

The online version contains supplementary material available at 10.1186/s40249-025-01302-y.

## Background

Schistosomiasis, one of the most widespread neglected tropical diseases (NTDs), is caused by blood-dwelling parasitic flatworms from the genus *Schistosoma*. The primary species infecting humans are *Schistosoma mansoni*, *S. japonicum*, and *S. haematobium*, while *S. guineensis*, *S. intercalatum*, and *S. mekongi* are less common [1]. Other species are associated with both wild and domestic animals, and increasingly attention is focusing on the possible interaction of schistosome species from a One Health perspective [2]. Schistosomiasis is endemic in 78 low- and middle-income countries across tropical and subtropical regions of the World, affecting over 250 million people worldwide [3]. The rationale for maintaining a diverse, field-collected archive of schistosomiasis-relevant samples and the importance of integrating genomic and epidemiological data in understanding schistosomiasis has been reviewed elsewhere [4, 5]. Briefly, excellent resources to support schistosome research with live parasite material, such as the US National Institutes of Health (NIH-NIAID) Schistosomiasis Resource Center, have existed for many years [6]. In consequence, the parasite strains they supply are the product of long-term laboratory passage. For example, one of the standard isolates, *S*. *mansoni* strain NMRI, was isolated in the 1940s. A parasite population isolated for 80 years, with a generation time in the lab measured in weeks cannot be used to address research questions related to evolution and diversity in the field. The Schistosomiasis Collection at the Natural History Museum (SCAN) was created to help fill this gap.

SCAN is a genetic/genomic resource focused on schistosomes and their intermediate snail hosts that originated in 2010, part funded by the Wellcome Trust [7]. It is based at the Natural History Museum (NHM) in London, which houses one of the largest biological collections in the world, comprising around 80 million specimens [8]. SCAN applies collections management principles to a working research collection, thereby making samples visible and available to the wider research community. The collection grew in close association with SCORE, the Schistosomiasis Consortium for Operational Research and Evaluation [9], and built on an existing legacy collection of schistosomes and snails in the field through NHM’s separate research activities over many decades. SCAN collections therefore consist of both recent field-collected specimens (mostly larval schistosomes and intermediate freshwater snail hosts); and legacy material (short-term lab passaged schistosomes and snails historically collected from the field). A scoping review was undertaken to assess the usage of the Collection on schistosomiasis research in a structured way, to assess the impact and reach of the SCAN Collection.

## Methods

### Search strategy and inclusion criteria

The literature search was conducted using Google Scholar and Scopus. Search terms included: SCAN, the schistosomiasis collection at the NHM, and schistosomiasis. Separate searches were carried out for citations of the publication which originally laid out the scope of the SCAN collection [7]. The date of last search was the 1st of September 2024. Studies were included if they met the following criteria: the SCAN publication was cited, and/or if the SCAN Collection was utilised in the work. Studies were excluded if they were duplicates, e.g. a preprint of a peer-reviewed work later published. A contextual search, i.e. the wider schistosomiasis research field, was not conducted given the extent of the literature.

### Data extraction and analysis

Data extracted from studies included: year of publication, authors, whether and how the SCAN Collection was used in the work, and type of collection, i.e. legacy (mostly lab-passaged schistosomes) or recent (field-collected) material. All publications were categorised by subject area/topic. Other details collected included publisher/journal, publication type, e.g. thesis/article/preprint. Overall quality was assessed by total citations of each publication, along with the journal quartile ranking (derived from Clarivate *Web of Science,* copyright Clarivate 2024, all rights reserved).

Key applications of the SCAN Collection were assessed in a qualitative fashion to summarise the focus of studies. Summary data on the SCAN Collection and usage of material were compiled, both for recent and legacy collections, to provide an overview of the collections data itself (see scan.myspecies.info and the NHM data portal, https://data.nhm.ac.uk). All data were analysed and processed in R, version 4.0.2 (https://www.r-project.org/) “Taking Off Again”, R-Foundation, Vienna, Austria [10], and Microsoft Excel 365 (version 2409, Microsoft, Redmond, USA). All mapping was conducted in Quantum GIS (QGIS, version 3.10, Coruña. QGIS.ORG, Gossau, Switzerland) [11].

## Results

### Bibliometric results

**Fig. 1:**
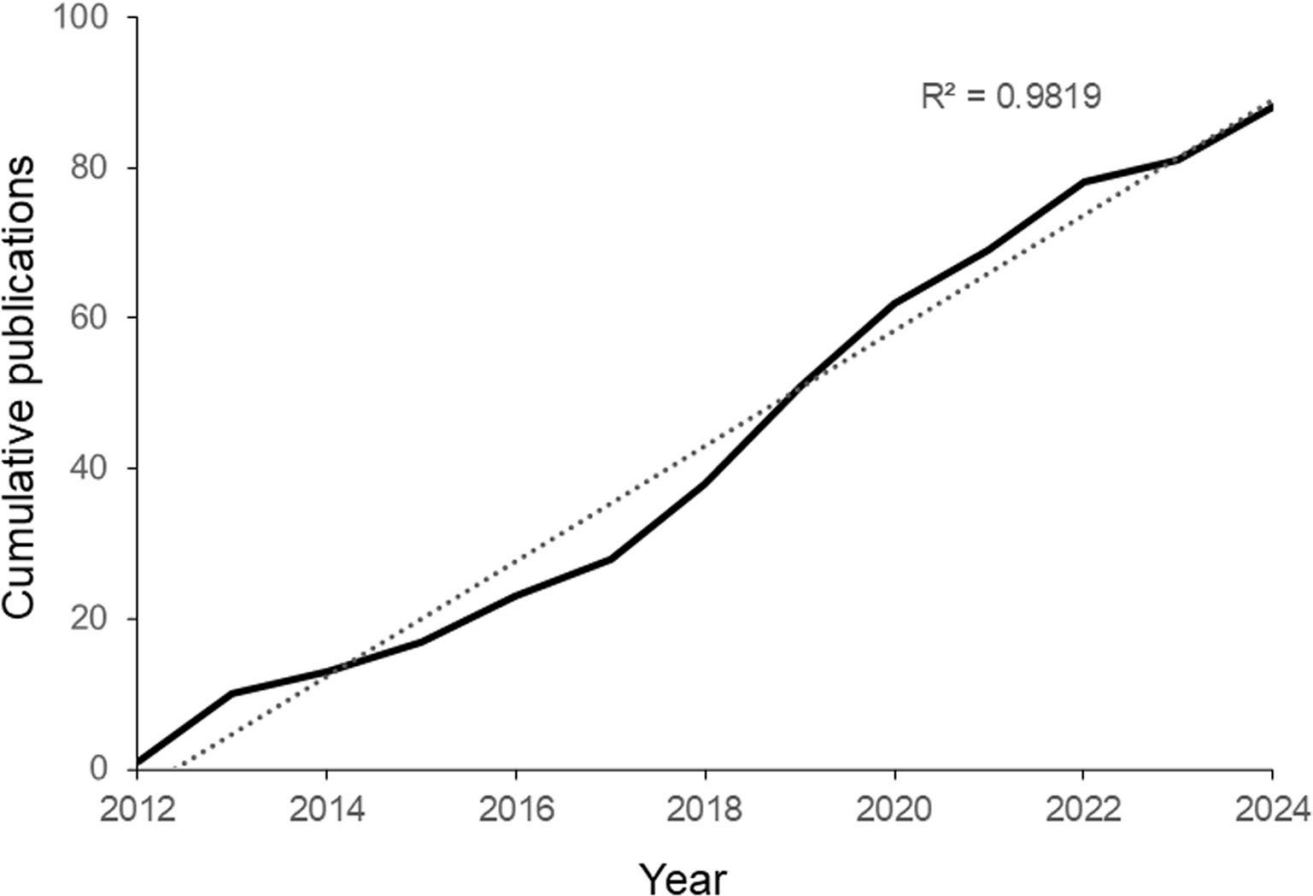
Cumulative publications based on SCAN 2012–2024. Cumulative publications fulfilling the search criteria are plotted as a solid line. The linear regression line is plotted as a dotted line, coefficient of determination R^2^ = 0.98. *SCAN* Schistosomiasis Collection at the Natural History Museum

The search found 88 papers in total as citations of the SCAN publication, direct citations of the Collection or both (Table 1). Of these works, 81 were peer-reviewed articles, four theses, two preprints and a field guide. Articles were published in 37 different titles, the majority being parasitology or public-health journals, the remainder (16) other disciplines or more general biology/science publications (see supplementary data 1). Overall, three-quarters of the publications citing the paper utilised the Collection (66/88). The remainder were review papers. Paper categories were as follows: epidemiology/operational research; field guide; genomics/genetics/phylogenetics of schistosomes/of intermediate host snails; molecular diagnostics; review; intermediate host snail ecology/mapping/modelling. For proportions of publications by category, 23% genetics/phylogeny/species identification, 25% genomics/population genomics (including molecular-functional applications), 18% molecular diagnostics, 10% snail ecology/mapping/modelling, 19% reviews and 6% operational research/epidemiology/field guide (see Table 1, supplementary data 1). Half the publications focussed on schistosomes, 16% both on schistosomes and intermediate host snails, 9% on snails only, the remainder not applicable, e.g. review articles (Table 1). The 88 publications were cited a total of 3625 times (range 0–401 citations per publication, median 29 citations). The majority of journal publications (96%) were published in journals with a Q1 (66%) or Q2 (30%) quartile ranking. Publications over the period 2012–2024 appeared consistently over time, with a mean of 7 per year (minimum of 1 in 2012, peaking at 13 in 2019; Fig. 1).

**Table 1:** Publications utilising the SCAN Collection in research or citing Emery et al. 2012 [7]

Year	Publication	Focus	Category	Collection?
2012	Emery et al. [7]	sch/sn	Review	–
2012	Webster et al. [12]	sch	Genomics/population genetics	y
2013	Allan et al. [13]	sn	Molecular diagnostics	y
2013	Cnops et al. [14]	sch	Molecular diagnostics	y
2013	Glenn et al. [15]	sch	Genetics/phylogeny	y
2013	Huyse et al. [16]	sch	Genomics/population genetics	y
2013	Kane et al. [17]	sch/sn	Molecular diagnostics	y
2013	Lopes et al. [18]	sch	Genomics/molecular-functional	–
2013	Sealey et al. [19]	sch	Genetics/phylogeny	y
2013	Stothard et al. [20]	sch	Review	–
2013	Utzinger et al. [21]		Review	–
2014	Knopp et al. [22]	sch	Operational research/epidemiology	–
2014	Moser et al. [23]	sn	Snail ecology/mapping/modelling	y
2014	Stothard et al. [24]	sch	Review	–
2015	Gleichsner et al. [4]	sch	Review	–
2015	Rosser et al. [25]	sch	Molecular diagnostics	y
2015	Van den Broeck et al. [26]	sch/sn	Genetics/phylogeny	y
2015	Webster et al. [27]	sch	Genetics/phylogeny	y
2016	Crellen et al. [28]	sch	Genomics/population genetics	y
2016	Easton [29]		Soil transmitted helminths	–
2016	Knopp et al. [30]	sch	Operational research/epidemiology	–
2016	Léger et al. [31]	sch	Genetics/phylogeny	y
2016	Nussbeck et al. [32]		Review	–
2016	Pennance et al. [33]	sch/sn	Snail ecology/mapping/modelling	y
2017	Abbasi et al. [34]	sch	Genomics/population genetics	y
2017	Allan et al. [35]	sch/sn	Snail ecology/mapping/modelling	y
2017	Crellen [36]	sch	Genomics/population genetics	y
2017	Gouvras et al. [37]	sch/sn	Snail ecology/mapping/modelling	y
2017	WHO [38]	sn	Field guide	–
2018	Abe et al. [39]	sn	Genetics/phylogeny	y
2018	Anderson et al. [40]	sch	Genomics/population genetics	y
2018	Boon et al. [41]	sch	Genomics/population genetics	y
2018	Booth & Clements [42]	sch/sn	Review	–
2018	Lawton et al. [43]	sn	Genetics/phylogeny	y
2018	Le Clec’h et al. [44]	sch	Genomics/molecular-functional	y
2018	Pennance et al. [45]	sch	Genetics/phylogeny	y
2018	Poulton & Webster [46]	sch	Molecular diagnostics	y
2018	Sene-Wade et al. [47]	sch	Genetics/phylogeny	y
2018	Tian-Bi et al. [48]	sch/sn	Operational research/epidemiology	y
2019	Boon et al. [49]	sch	Genetics/phylogeny	y
2019	Catalano et al. [50]	sch	Molecular diagnostics	y
2019	Chevalier et al. [51]	sch	Genomics/population genetics	y
2019	Doyle et al. [52]	sch	genomics/molecular-functional	y
2019	Harmon et al. [53]		Review	–
2019	IHGC [54]	sch	Genomics/molecular-functional	y
2019	Oey et al. [55]	sch	Genomics/population genetics	y
2019	Papaiakovou et al. [56]		Soil transmitted helminths	–
2019	Platt et al. [57]	sch	Genomics/population genetics	y
2019	Rabone et al. [58]	sch/sn	Snail ecology/mapping/modelling	y
2019	Rostron et al. [59]	sch	Molecular diagnostics	y
2019	Tian-Bi et al. [60]	sch	Genetics/phylogeny	y
2019	Wood et al. [61]	sch/sn	Snail ecology/mapping/modelling	y
2020	Allan et al. [62]	sn	Review	–
2020	Archer et al. [63]	sch	Molecular diagnostics	y
2020	Catalano et al. [64]	sch	Genetics/phylogeny	y
2020	Colley et al. [65]	sch/sn	Review	–
2020	Colley et al. [9]	sch/sn	Review	–
2020	Keller et al. [66]	sch	Molecular diagnostics	–
2020	Léger et al. [67]	sch	Genetics/phylogeny	y
2020	Pennance [68]	sch/sn	Genetics/phylogeny	y
2020	Pennance et al. [69]	sch/sn	Snail ecology/mapping/modelling	y
2020	Pennance et al. [70]	sn	Molecular diagnostics	y
2020	Webster et al. [71]	sch	Review	–
2021	Berger [72]	sch	Genomics/population genetics	y
2021	Berger et al. [73]	sch	Genomics/population genetics	y
2021	Halili et al. [74]	sch	Molecular diagnostics	y
2021	Le Clec’h et al. [75]	sch	Genetics/phylogeny	y
2021	Rey et al. [76]	sch	Genomics/population genetics	y
2021	Tallam et al. [77]	sch/sn	Snail ecology/mapping/modelling	y
2021	Thompson et al. [78]		Review	–
2022	Berger et al. [79]	sch	Genomics/population genetics	y
2022	Landeryou et al. [80]	sch	Genomics/population genetics	y
2022	Lund et al. [5]	sch	Review	–
2022	Mesquita et al. [81]	sch	Molecular diagnostics	y
2022	Miswan et al. [82]		Soil transmitted helminths	–
2022	Moser et al. [83]	sch/sn	Operational research/epidemiology	y
2022	Pennance et al. [84]	sch/sn	Genetics/phylogeny	y
2022	Stroehlein et al. [85]	sch	Genomics/molecular-functional	y
2022	Webb et al. [86]	sch	Molecular diagnostics	y
2023	Cherkaoui et al. [87]	sch	Molecular diagnostics	y
2023	Senghor et al. [88]	sch/sn	Genetics/phylogeny	y
2023	Trippler et al. [89]	sch	Review	–
2024	Ajakaye et al. [90]	sch	Genetics/phylogeny	y
2024	Andrus et al. [91]	sn	Genetics/phylogeny	y
2024	Archer et al. [92]	sn	Molecular diagnostics	y
2024	Berger et al. [93]	sch	Genomics/population genetics	y
2024	Donnelly et al. [94]	sch	Molecular diagnostics	y
2024	Platt et al. [95]	sch	Genomics/population genetics	y
2024	Stelbrink et al. [96]	sn	Genetics/phylogeny	y

“Focus” refers to the focus of each paper on schistosomes (sch), snails (sn) or both (sch/sn). More general reviews or papers on non-snail/schistosome topics are left blank. Papers resulting from work that directly used SCAN are marked “y” in the “Collection?” column (otherwise marked “−”)

### Overview of collections data

#### Spatial, temporal and taxonomic representation/coverage

SCAN covers recent field-collected specimens, both schistosome larval stages (mainly stored on FTA cards: formerly Whatman, now manufactured by QIAGEN, Venlo, Netherlands), and freshwater snail intermediate hosts (stored in ethanol); and a legacy collection of adult worms recovered after laboratory passage and frozen, maintained in liquid nitrogen. The legacy material also includes an extensive collection of field-caught African freshwater snails preserved in spirit or as dry shells. The recent (field-collected) specimens comprise close to half a million individual life stages of schistosomes, housed on approximately 10,000 FTA cards, and legacy collections of over 3000 schistosome specimen lots of adult worms, housed in liquid nitrogen. Snail holdings include recent collections of over 80,000 specimens, stored in ethanol, and legacy collections of over 130,000 specimens, all field-collected. The collection also includes over 14,000 DNA vouchers (mainly of *S. mansoni* and *S. haematobium*), stored at − 80 °C.

Almost all trematode species within the collection are schistosomes, in particular *S. haematobium* and *S. mansoni,* with half of the known species of schistosomes represented (Table 2). Non-schistosome flukes are also represented in the collection, for example *Paramphistomum*, *Fasciola*, *Plagiorchis* and unidentified trematodes. For freshwater snail collections, 26 genera are represented from recent collections, the majority being schistosome intermediate host snails, *Bulinus* and *Biomphalaria*. The legacy freshwater snail collections have much broader taxonomic coverage, with 110 genera represented.

**Table 2:** Summary of schistosome collections by species covering both legacy collections of laboratory-passaged schistosomes in liquid nitrogen and recent (field) collections on FTA/ethanol

Species	Legacy	Recent field collections			Miracidia	Total
	Adult worms*	Adult worms	Cercariae	Eggs		
*Schistosoma bovis*	359	213	8370	71	1130	9784
*Schistosoma curassoni*	79	30	335		941	1306
*Schistosoma guiniensis*	109	5				5
*Schistosoma haematobium*	550		8956	957	187,183	197,096
*Schistosoma intercalatum*	86					0
*Schistosoma japonicum*	25	955	17,120		1917	19,992
*Schistosoma mansoni*	1060	122	31,673	245	141,280	173,320
*Schistosoma margrebowiei*	146					0
*Schistosoma mattheei*	117		240			240
*Schistosoma rodhaini*	60		34			34
*Schistosoma* sp.	879		27,671	35	22,569	50,275
*Schistosoma spindale*	3					0
Trematoda			12,264		16	12,280
	3473	1325	106,663	1308	355,036	464,332

^*^Specimen lots of adult worms from legacy collection

Both recent and legacy collections show a broad range of spatial and temporal coverage, encompassing 17 countries from which schistosomes have been sampled, rising to 27 when including snail collections. Recent collections from the SCORE programme [65], which comprise a large proportion of recent SCAN specimens, have a narrower focus, e.g. mainland Tanzania, Zanzibar and Niger. However, these collections have temporal depth being longitudinal multi-year surveys, reflecting project aims to undertake population genetics and to assess the impact of drug pressure on populations. Overall, SCAN shows broad spatial coverage given the range of projects, including Angola, Cameroon, China, Cote d'Ivoire, Eswatini, Ethiopia, Kenya, Liberia, Madagascar, Malawi, Namibia, Niger, Senegal, Sudan, Tanzania, Uganda and Zambia, 1646 georeferenced sites in total, ranging from a single site up to 829 per country (Tanzania). The majority of holdings are from sub-Saharan Africa, but some collections and species from south-east Asia are represented, particularly *S. japonicum* from China (Table 2). The main countries represented are Niger and Senegal in West Africa, and Tanzania (both mainland and Zanzibar) and Uganda in East Africa (Table 2).

Recent collections of schistosomes (collected and stored on FTA cards or liquid nitrogen) and snails (stored in molecular grade ethanol) cover 1997 to 2020. Legacy schistosome collections (lab passaged and stored in liquid nitrogen) and snails (stored in industrial methylated spirit or dry) range from 1933 to 2007, therefore overlapping in time. The most accessed collections are schistosomes, both legacy adult worms (stored in liquid nitrogen) and recent (FTA/ethanol preserved), the former in particular have been used for genomics studies (see following section). The usage of the collections shows a wide range of work supported, e.g. from small-scale projects on snail phylogenetics to broad-scale studies of parasite genomics.

### Contributions to key narratives in recent schistosome research

SCAN has partnered with numerous projects and organisations but standardised the collection of larval schistosomes wherever possible. A full methodology is provided below, expanding previously published details [44]. Key SCAN research applications include phylogeography, genomic analysis of hybridisation, genomics of drug resistance and selection, all possible through provision of legacy, recent and rare material.

#### Sample collection and preparation methods used by SCAN and partners

##### FTA storage

Use of FTA cards ((QIAGEN) as a collection/storage medium for larval schistosomes originated with Gower et al. [97], and has been reported many times since [98–101]. FTA cards are a proprietary collection/storage format based on chemically treated cards, whereby cells pipetted onto the cards are lysed on contact and the DNA released is stabilised within the card matrix, which, after drying, can be stored at room temperature. Eliminating the need for a cold-chain and additional chemical fixatives combined with portability, makes the method an attractive one for field use, with the additional benefits of long-term storage at ambient temperature, and efficiency of space. The entire SCAN ambient collection, of close to half a million individual schistosome larvae collected on FTA cards, fits in a single museum collections cabinet (approximately 2 m × 1.5 m). The methods used defer complex procedures from the field environment to the laboratory, and separating individual parasites rather than combining into pools maximises the utility of the Collection e.g. for population genetic analysis, where understanding segregation of alleles among individuals is essential, or for whole genome sequencing, where assembly is facilitated without population-level variation.

The original method involved hatching of schistosome eggs in filtered water and transfer of each miracidium to the card in a small volume (2–3 µl) using a micropipette [97]. Essentially the same technique can be used for cercariae, by placing the snail into clean water, allowing cercariae to emerge and pipetting each individually onto the card with an equivalent water volume as for miracidia. The DNA on the card can then be prepared as a template for PCR by punching out the area of the card containing the DNA using a 2.0 mm Harris micro-punch, followed by multiple washes in FTA purification reagent and TE buffer, according to manufacturer’s instructions. The dried paper punch containing the DNA can then be used as the template in a PCR. A key drawback of the original method is that only a single PCR can be carried out per cercaria or miracidium [100]. Refinements to all steps in the collection and extraction methods have since allowed the DNA to be used for multiple applications and generated genome-level data from single larvae, as summarised below [27, 44, 52].

#### Detailed larval schistosome collection and downstream preparation protocols

##### Preparation and transfer of miracidia

**Fig. 2:**
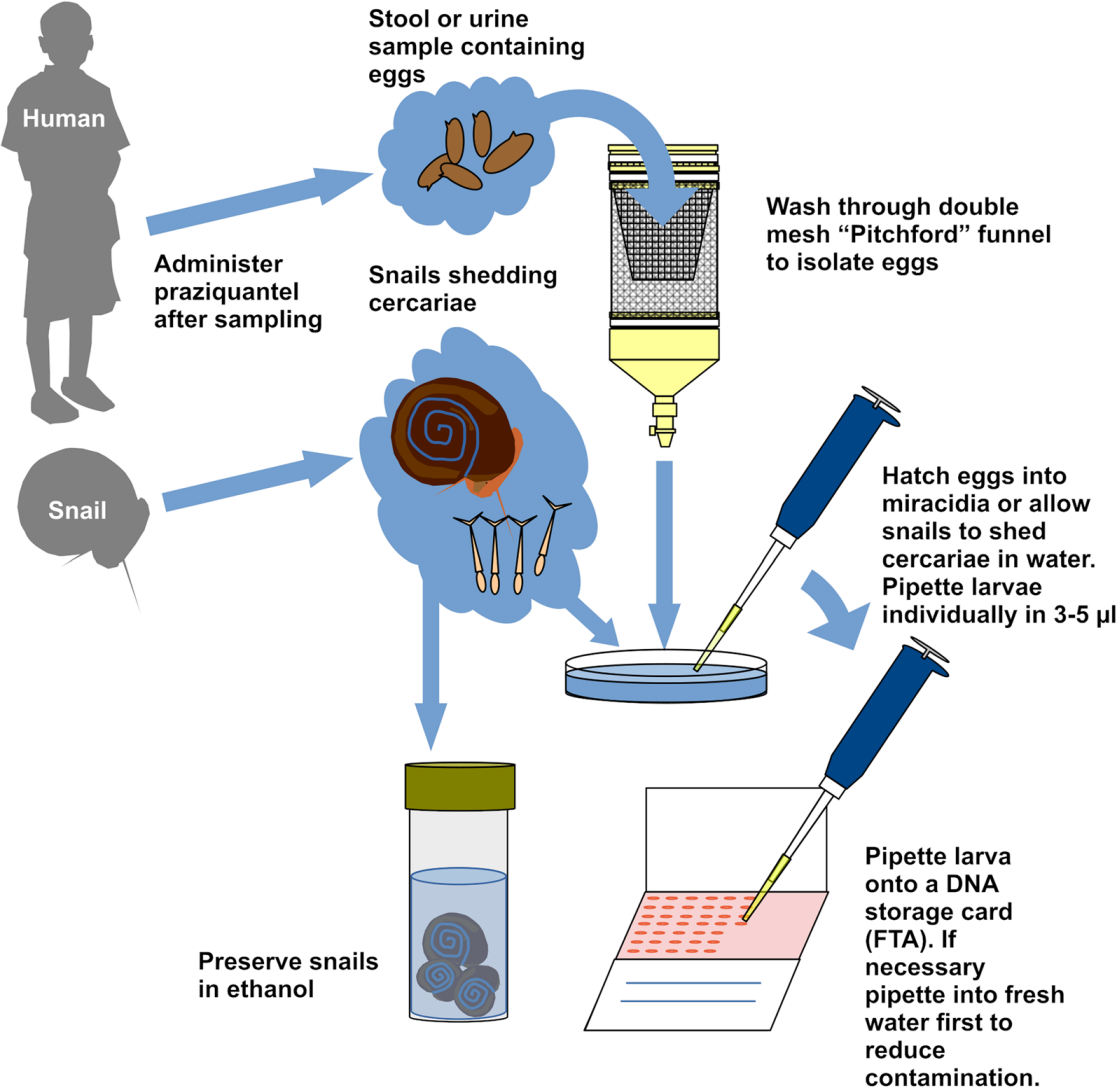
Diagram of steps involved in collecting schistosome larval stages onto DNA storage cards

The method used for preparation of miracidia for most SCAN collections, including our collaborations with many independently-managed projects, was based on that of Visser and Pitchford [102], modified for portability (Fig. 2). Briefly, a stool sample of approximately 1 cm^3^ is rinsed through a metal sieve, 212 µm mesh (Endecotts, London, UK) using locally supplied bottled mineral water. This is then transferred to a “Pitchford” funnel, made up of a nylon 200 µm mesh inner bag and a 40 µm mesh outer sleeve attached to a funnel drained via a tap. Urine samples can be similarly transferred without the initial sieving. The stool/urine preparation is washed through with more mineral water and excess water drained out through the outer mesh until a small volume is retained in the funnel at the base of the outer sleeve. This is then transferred into a Petri dish (90 mm diameter, 15 mm depth) by opening the tap and the volume made up with clean water so that the dish is full to approximately three-quarter depth. Alternatively, urine samples can be filtered through a 20 µm PCTE filter (13 mm diameter) using a 10 ml syringe attached to a Swinnex filter housing (Merck-Millipore, Burlington, USA), components of the Schistosome Test Kit (Sterilitech, Auburn, USA). Eggs are retained on the filter which can then be placed in a Petri dish containing clean water as above.

Placed under light, the Petri dish is then left for 30 min to two hours to allow miracidia to hatch from the eggs. Miracidia migrate clear of co-filtered debris and can be individually pipetted in a volume of 3 µl onto an indicating FTA Classic card (QIAGEN). Contamination with faecal/urine material and bacteria can be reduced by a ‘double wash’, pipetting first into a small volume of clean water (approximately 10–20 µl) in a second dish, repeating this step, and then from there transferring the miracidia to the card. Cercariae can also be transferred from snails shedding in clean water to cards by pipetting in 3 µl. Measurements for a Pitchford funnel are provided in supplementary Fig. 1.

##### Preparation of larval schistosomes on FTA cards for further genetic analysis

**Fig. 3:**
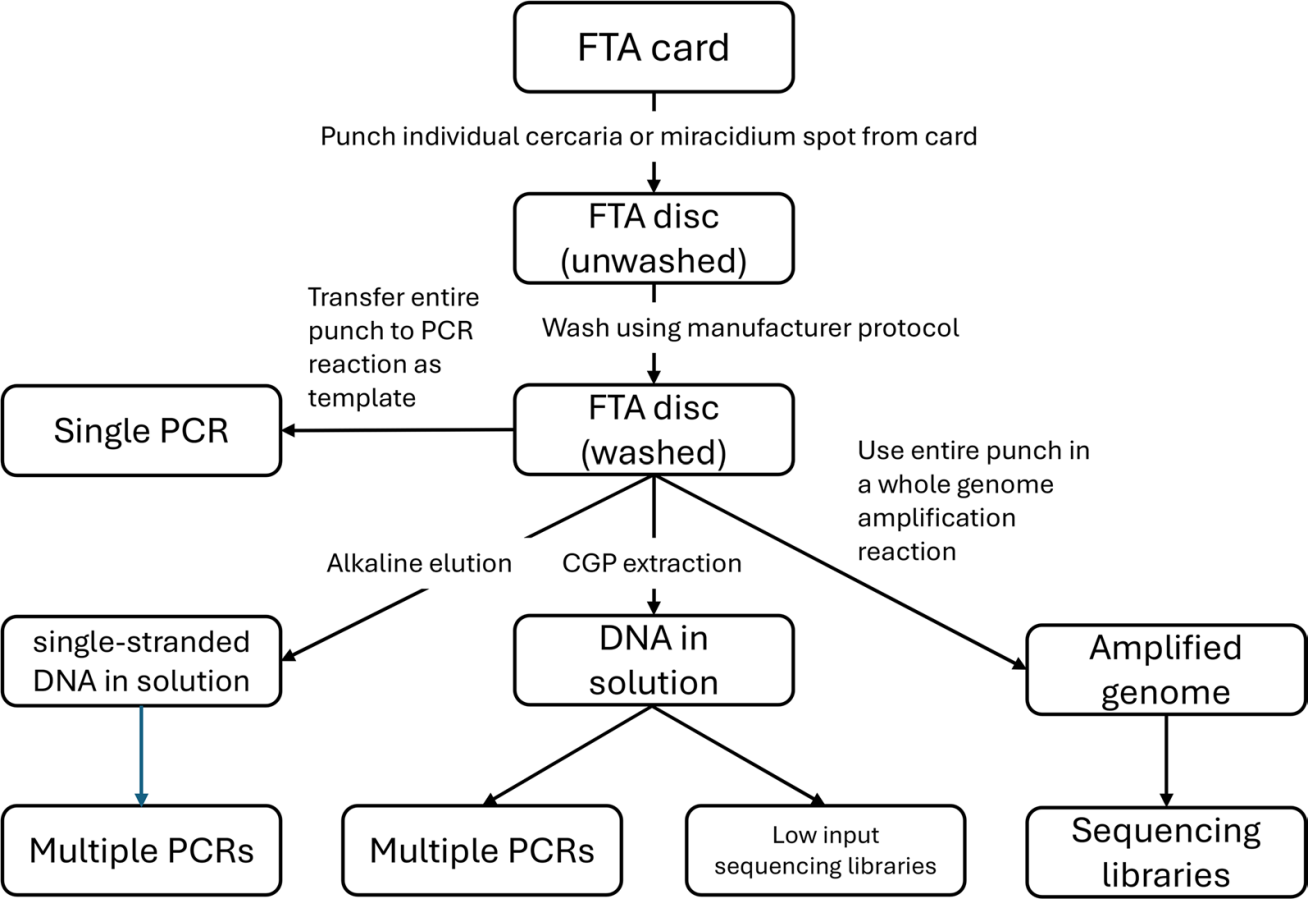
Flow diagram of methods for processing schistosome larval stages collected on DNA storage cards. Methodological details are given in the text

Using the standard manufacturer’s protocol involves taking a punch from the card using a 2 mm UniCore manual punch (QIAGEN), washing three times in 200 μl of QIAcard FTA Wash Buffer (QIAGEN), then 200 μl of TE^−1^ Buffer (10 mmol/l Tris-HCI, 0.1 mmol/l EDTA, pH 8.0), before drying and using the punch as a template within a polymerase chain reaction (PCR). There are numerous refinements to the procedure to allow more flexibility, increasing the potential use of each schistosome larva beyond a single PCR, several of which are discussed by Doyle et al. [52]. Examples below have been used for preparation of samples from SCAN (Fig. 3):

1. Alkaline elution [27] involves releasing the DNA by denaturation in 14 µl of 0.1 mol/l NaOH, 0.3 mmol/l EDTA followed by reducing the pH to just above neutral using 26 µl of 0.1 mol/l Tris–HCl, pH 7.0. The resulting eluate can be used directly as PCR template, using around 3 µl per 25 µl reaction.
2. CGP extraction [103] involves a 1 h incubation of the FTA punch in a lysis buffer consisting of 1.25 μg/ml of protease reagent (QIAGEN) in Tris HCl pH 8.0, 0.5% Tween 20, 0.5% NP40) followed by heat inactivation of proteinase at 75 °C. The eluate is then purified using Agencourt AMPure XP beads (Beckman Coulter, Brea, USA) and used to make sequencing libraries.
3. Whole genome amplification [44] involves the manufacturer protocol to clean the punch (as given above), followed by whole genome amplification using the GenomiPhi V2 DNA amplification kit (Cytiva, Marlborough, USA) and cleaning with SigmaSpin sequencing reaction clean up (Sigma-Aldrich, St Louis, USA).

#### Examples of research applications

##### Species diversity

Two studies, using SCAN, lay out the framework for phylogeography of the two major African schistosome species [12, 104]. In a comparative survey of partial Cytochrome c Oxidase 1 (cox1) haplotypes of *S. mansoni* across sub-Saharan Africa, Webster et al. [104] found considerable diversity, both within and between individual human hosts, with haplotype lineages clustering along geographic lines. A similar study of *S. haematobium* showed a very different picture, with very little diversity throughout Africa, except for a second cluster of haplotypes found in coastal Kenya, the Zanzibar/Mafia islands archipelago and Mauritius [12]. A recent study comparing genomes of 219 *S. haematobium* and *S. bovis* samples, the majority accessed via SCAN, brings new clarity to the north/south genetic differentiation between *S. haematobium* populations [95].

##### Hybrids

Inter-species hybridisation has often been reported for schistosomes, particularly the *S. haematobium* clade between *S. haematobium* and *S. bovis* [16, 105–107]. Many studies identified likely hybrids through discordance between mitochondrial and nuclear gene sequences [16, 90, 108]. However, while these methods provide indicators of hybridisation, they do not tell us whether hybridisation is ancient or contemporary, or the consequences of introgression. Further studies required development of a combination of molecular techniques allowing genomic study of the larval stages alongside field-collected samples being made available through SCAN. The first publication of a *S. bovis* genome using DNA from a laboratory-passaged parasite originally collected from Tanzania, maintained at NHM and stored in SCAN, allowed direct genome comparison between *S. bovis* and the reference *S. haematobium* genome originating from an Egyptian isolate [55]. While the mean similarity between the genomes is high, around 97%, there were regions up to several hundred kilobases in length that approached 100% similarity, indicating that the Egyptian isolate of *S. haematobium* incorporated and retained parts of the *S. bovis* genome an unknown number of generations beforehand.

Meanwhile, new techniques applied to individual schistosome miracidia preserved on FTA cards maintained in SCAN have enabled full genome/exome sequencing of this material [44]. These approaches allowed populations of *S. haematobium* miracidia from the Zanzibar archipelago and Niger to be compared, confirming evidence of ancient hybridisation with *S. bovis* (estimated 108–613 generations previously) for the latter population, but no evidence of hybridisation for Zanzibar. The consequences of introgression could also be explored, with an introgressed invadolysin gene, likely involved in mammalian host interactions, showing the strongest signature of directional selection [57]. The genetic diversity of *S. haematobium*, its regional introgression with *S. bovis*, the ancient nature of the hybridisation and the selection of introgressed alleles have been examined in a new whole-genome pan-African study using SCAN archives [95]. This adds weight to the conclusions of the previous work and provides the basis for further studies into introgressive adaptation.

The replacement by *S. haematobium* of the rarer *S. guineensis* through a process of introgressive hybridisation was observed in real time from the late 1960s until the late 1990s around Loum, Cameroon [105]. The event pre-dates the advent of genome-level sequencing, but recent analysis of schistosomes from the region collected between 1990 and 1998 and cryopreserved in SCAN reveal the contemporaneous nature of the interaction, with up to 50% of alleles attributed to *S. haematobium* contrasting with an island population of *S. guineensis* from Sao Tomé with no introgression [80]. The high proportion of the genome still originating from *S. guineensis* as late as 1998 is interesting, given that urogenital schistosomiasis had replaced intestinal schistosomiasis in the region by this time. Given that the *S. haematobium* analysed in the study may show evidence of the ancient hybridisation with *S. bovis*, described above, the final Cameroon population may have acquired introgressed alleles originating from two other species.

Finally, using a cryopreserved isolate of *S. curassoni* stored in SCAN since 1993, Berger et al. [79] constructed an improved reference genome of the species. This permitted comparisons of livestock schistosomes (*S. curassoni* and *S. bovis*) from the *S. haematobium* clade collected in Senegal with the assistance of SCAN. The samples, a combination of adult worms and miracidia collected from abattoirs, again demonstrated contemporary rather than ancient hybridisation occurring between these two species in this location.

While improvements in DNA sequencing technology and the availability of archive samples came together to improve our understanding of interspecies hybrids, there is still much to learn about whether hybrids have an ongoing, current impact on schistosomiasis control, or whether their significance is in understanding the history and evolution of the parasite. The latter may provide important information for interventions if introgression followed by selection incorporates regional genotypic/phenotypic variants or reduces genetic diversity of the parasite, affecting (for example) the diversity of vaccine candidate antigens and standing variation of genes associated with drug resistance.

##### Drug resistance

The anthelmintic drug praziquantel remains the keystone of schistosomiasis control programmes [109]. Until recently, a lack of understanding of its precise target within schistosomes precluded any genetic surveillance of possible drug resistance markers. However, an important proof-of-principle study investigated a second drug, oxamniquine, previously used in South America to treat infection with *S. mansoni* [51]. In this case, the mechanism of resistance is well understood, and can be identified through characteristic mutations of the schistosome sulfonotransferase (*SmSULT-OR*) gene. Using miracidia samples of *S. mansoni* from Tanzania, Niger and Senegal held in SCAN, along with samples separately collected from Oman and Brazil, the study revealed that resistance alleles are relatively abundant in Africa and Oman in addition to Brazil. Oxamniquine resistance therefore resulted from selection of resistance alleles already present in natural populations. More recent studies have now identified the *S. mansoni* transient receptor potential melastatin channel (*SmTRPM*) as praziquantel’s target [75, 110]. The same panel of genomes revealed a single sample from Oman with a mutation of *SmTRPM* present as a heterozygous allele, which would lead to truncation and loss of function. Therefore, potential praziquantel resistance alleles are present in natural populations, albeit rarely.

Building on these initial studies, genomic surveillance studies should link to control programmes, where compliance with, and coverage of, MDA and treatment history is recorded alongside clinical outcome and additional information associated with the genome data. A recent example is a large study sampling schistosome miracidia in schools in Uganda, collected in association with Unlimit Health (previously the Schistosomiasis Control Initiative) with archiving and processing support from SCAN [73]. This study found no evidence of a resistant *S. mansoni* population contributing to reduced drug efficacy. However, a new study using archived miracidia from SCAN and other collections identified four variants in natural populations that affect praziquantel susceptibility from predicted functional profiling [93].

## Discussion

Theodor Bilharz discovered human schistosomes in 1851, but it was well into the twentieth century before their life cycle was fully understood [111]. Acrimonious debate among the senior academics of the time about whether transmission was direct or indirect can only have been the result of adopting entrenched positions in the absence of evidence. Today’s research community should not be complacent about avoiding similar mistakes. NTD research may be at particular risk since sampling is challenging, funding is limited, and there may be pressure for hypotheses to influence models and policy prematurely. In the NTD world therefore, the critical strategic importance of biospecimen repositories may be to provide access to material for basic ground truthing. In SCAN’s case, this has often been a first step that initiates further sampling programmes.

SCAN demonstrates the benefits of creating collections beyond the capacity of any individual project, introducing new researchers into this field by providing access to samples, and allowing longitudinal population-level comparisons at scale. The collection grew by providing support to associated fieldwork projects, upscaling and standardising collections so that they could be archived for additional study. SCAN has therefore been able to support a diverse range of projects, from large-scale population genetics studies alongside drug treatment programmes, to small-scale intermediate host snail phylogenetics studies. The benefits of the approach are clear from the applications and outputs given above. The experience also highlights the challenges discussed below.

SCAN occupies an unusual position as a genetic/genomic resource focused on schistosomes and their snail hosts, rather than following a more typical human-centred biobank model. This focus has been advantageous in building a large, diverse collection, representing the genetic diversity of a highly complex combination of parasite species including introgressed forms. However, linking samples to participant history, participant samples (e.g. serum, blood, stool, urine) and clinical outcome data at large scale are also important features of clinical population biobanks not incorporated into SCAN. Such combinations enable retrospective interrogation of genomes, analytes and data to understand underlying correlations with disease [112]. Non-clinical “biodiversity” biobanks, often with a diversity of sample types, can face a multitude of challenges, including lack of standardisation, a paucity of associated data, and ill-defined purpose [113]. SCAN has attempted to overcome these potential pitfalls by focusing sampling on schistosomes and snails, standardising collections where possible and incorporating what data are available. Access to field collected specimens for schistosomiasis research has been very limited indeed, making *any* genetically diverse sample sets and associated geographic information very valuable for research purposes. By providing these at scale, SCAN has made an important contribution to schistosomiasis research. The ideal schistosome biobank, however, would also incorporate patient samples and data on history and clinical outcomes (necessitating careful consideration of patient confidentiality and a robust informed consent process [114, 115]).

Sustainability and long-term funding are significant problems for biobanks [116]. A decade of funding by the Wellcome Trust between 2011 and 2021 allowed the establishment and rapid expansion of SCAN, with staffing to support collection projects, archive samples and data, respond rapidly to requests, and facilitate research in general. The programme demonstrated that moderate investment in a bioarchive can bring new researchers into the field and develop innovative avenues of research. Regrettably, SCAN is currently unfunded, the NHM maintains holdings but can neither expand nor support new collection initiatives.

SCAN is limited to providing preserved specimens, whereas conducting functional studies requires live snails and schistosomes. Such resources are available from individual research groups and resource providers such as the Schistosomiasis Resource Center [6]. A UK initiative between NHM and the London School of Hygiene and Tropical Medicine (SSR: Schistosome Snail Resource [117]) currently provides similar live material, but with the same fixed-term funding scheme used to support SCAN. This highlights a wider problem in science funding, a disconnect between the short-term timescales of funding phases, and long timescales of research and collections care [118]. The situation for SCAN has been ameliorated somewhat by its location at the Natural History Museum in London, which has a track-record in long-term collections management as a core responsibility. However, without additional funding, the long-term viability especially of the non-liquid nitrogen collection may be reduced. For example, curation activities that enhance long-term stability such as re-packing FTA cards in vacuum storage after use and re-spiriting snails in ethanol cannot be performed at ideal frequency without the additional staff.

### Nagoya protocol

The Nagoya Protocol to the Convention on Biological Diversity has been in force since October 2014. The protocol addresses access to genetic resources and the fair and equitable sharing of benefits arising from their utilization, but the past decade has seen extensive criticism of this framework for not meeting its objectives [119–121]. For example, some national implementations of the Nagoya Protocol have been overly restrictive, hampering research even by in-country researchers, and in some cases, this has led to revision of domestic access and benefit sharing (ABS) legislation [118]. The bilateral structure of Nagoya necessitates navigation of the legislation country-by-country, which has its challenges and inherent inefficiencies, including poor resourcing of national focal points in some countries. Recently, there has been a trend towards a more multilateral, harmonised approach towards benefit sharing which may provide a path forward [121].

Improving capacity in-country is key to implementing the objectives of the Nagoya Protocol. The publications citing SCAN illustrate reasonably good representation of authors from sub-Saharan Africa/LMIC overall (supplementary data 1), but few in first, last or corresponding author position. The research based on the Collection is highly collaborative, but more needs to be done to facilitate in-country-led research. This need has been discussed elsewhere, including specifically for sub-Saharan Africa [5, 122–124]. Sequencing technologies have significantly advanced since the launch of SCAN in 2011 and the Nagoya Protocol entering into force in 2014. An ideal future genomic surveillance scheme would be located in African institutions, which would generate genome data at source.

### Future directions

SCAN contains samples across a broad geographic and temporal range, providing specimens that can be used to assess large scale variation. SCAN may serve as the starting point for further work requiring more focused collection. In some cases, however, SCAN collections have been sufficiently intensive to create sample/data sets that can investigate genetic change over time. For example, specimens collected over several years as part of the Zanzibar Elimination of Schistosomiasis Transmission (ZEST) [84, 89] are now being sequenced to search for genetic markers of drug resistance. As whole genome sequencing costs reduce, the emphasis on storage should change to direct genomic surveillance, reducing the necessity for longer term sample retention. Ideally, genomic surveillance should be incorporated into control intervention monitoring and evaluation efforts by sequencing a proportion of the samples collected. SCAN’s model of standardising methods across different studies and providing a skilled team deployable at multiple sites provides an excellent example of how such work could be undertaken efficiently.

## Conclusions

The narrow limits of this review, focusing solely on the literature surrounding SCAN, demonstrate the utility of this single sampling initiative in enabling research into key areas of schistosome biology. The samples and accompanying data it provided represent raw materials whose scarcity was a limiting factor impeding genetic studies of this parasite. SCAN also assisted collecting activities of other projects through collaboration, standardized methodology, and provided access to samples for laboratory-based scientists without field experience. The resulting studies, including investigations of zoonoses, hybridisation and drug resistance, could not be undertaken without samples and data from the field. SCAN has demonstrated particular utility in providing material for studies into schistosome hybridisation that now show it to be a complex species- and location-specific phenomenon. While some of the intricacy of schistosome interactions and evolution have been revealed through these studies, the SCAN experience highlights the case for a more sophisticated approach to monitoring and evaluation, which would enable genomic surveillance of the parasite through structured sampling. While any field-collected material is beneficial for this, future collections should maximise links between samples and epidemiological data to track the impact of drug administration and other anthropogenic change on schistosome populations.

The limitation and short-term nature of resources to make this happen are familiar barriers, but unintended consequences of ABS legislation following the Nagoya Protocol risk making sample sharing even more problematic. The burden of ABS compliance falls on researchers with limited resources, and coordinated efforts at a higher level are needed to ensure that ABS encourages rather than inhibits collaboration. Sampling of schistosomes usually occurs at the point of their destruction via drug administration. Without sampling, these genetic resources are forever lost to science, and there are no benefits to share.

## Supplementary Information

Supplementary Material 1: Supplementary data 1: Publications.

Supplementary Material 2: Supplementary figure 1. Pitchford funnel dimensions.

## Data Availability

All data generated or analysed during this study are included in this published article and its supplementary information files.

## Abbreviations

ABS: Access and benefit sharing
LMIC: Lower middle income countries
NHM: Natural History Museum
NIH-NIAID: National Institutes of Health-National Institute of Allergy and Infectious Diseases
NTDs: Neglected tropical diseases
SSR: Schistosome Snail Resource
SCAN: Schistosomiasis Collection at the Natural History Museum
SCORE: Schistosomiasis Consortium for Operational Research and Evaluation
ZEST: Zanzibar Elimination of Schistosomiasis Transmission

## References

[1] McManus DP, Dunne DW, Sacko M, Utzinger J, Vennervald BJ, Zhou X-N. Schistosomiasis. Nat Rev Dis Primer. 2018;4:1–19. 10.1038/s41572-018-0013-8 30093684

[2] Díaz AV, Walker M, Webster JP. Reaching the World Health Organization elimination targets for schistosomiasis: the importance of a One Health perspective. Philos Trans R Soc Lond B Biol Sci. 2023;378:20220274. 37598697 10.1098/rstb.2022.0274 PMC10440173

[3] Hotez PJ, Alvarado M, Basáñez M-G, Bolliger I, Bourne R, Boussinesq M, et al. The global burden of disease study 2010: interpretation and implications for the neglected tropical diseases. PLoS Negl Trop Dis. 2014;8: e2865. 25058013 10.1371/journal.pntd.0002865 PMC4109880

[4] Gleichsner AM, Thiele EA, Minchella DJ. It’s all about those bases: the need for incorporating parasite genetic heterogeneity into the development of schistosome vaccines. PLoS Negl Trop Dis. 2015;9: e0003805. 26086424 10.1371/journal.pntd.0003805 PMC4472511

[5] Lund AJ, Wade KJ, Nikolakis ZL, Ivey KN, Perry BW, Pike HNC, et al. Integrating genomic and epidemiologic data to accelerate progress toward schistosomiasis elimination. Elife. 2022;11: e79320. 36040013 10.7554/eLife.79320 PMC9427098

[6] Lewis FA, Liang Y, Raghavan N, Knight M. The NIH-NIAID schistosomiasis resource center. PLoS Negl Trop Dis. 2008;2: e267. 18665228 10.1371/journal.pntd.0000267 PMC2480520

[7] Emery AM, Allan FE, Rabone ME, Rollinson D. Schistosomiasis collection at NHM (SCAN). Parasit Vectors. 2012;5:185. 22943137 10.1186/1756-3305-5-185 PMC3453491

[8] Miller CG, Brewer P, Carine M, Comerford G, Hardy H, Hart A, et al. Join the Dots: assessing a collection of 80 million items at The Natural History Museum, London. Mus Manag Curatorship. 2022;37:287–306.

[9] Colley DG, Jacobson JA, Binder S. Schistosomiasis Consortium for Operational Research and Evaluation (SCORE): its foundations, development, and evolution. Am J Trop Med Hyg. 2020;103:5–13. 32400343 10.4269/ajtmh.19-0785 PMC7351300

[10] R Core Team. R: A Language and Environment for Statistical Computing. R Foundation for Statistical Computing, Vienna, Austria. https://www.R-project.org. Accessed 1 Sep 2024.

[11] QGIS.org. QGIS Geographic Information System. QGIS Association. http://www.qgis.org. Accessed 1 Sep 2024.

[12] Webster BL, Emery AM, Webster JP, Gouvras A, Garba A, Diaw O, et al. Genetic diversity within *Schistosoma haematobium*: DNA barcoding reveals two distinct groups. PLoS Negl Trop Dis. 2012;6: e1882. 23145200 10.1371/journal.pntd.0001882 PMC3493392

[13] Allan F, Dunn AM, Emery AM, Stothard JR, Johnston DA, Kane RA, et al. Use of sentinel snails for the detection of *Schistosoma haematobium* transmission on Zanzibar and observations on transmission patterns. Acta Trop. 2013;128:234–40. 23318933 10.1016/j.actatropica.2013.01.003

[14] Cnops L, Soentjens P, Clerinx J, Van Esbroeck M. A *Schistosoma haematobium*-specific real-time PCR for diagnosis of urogenital schistosomiasis in serum samples of international travelers and migrants. PLoS Negl Trop Dis. 2013;7: e2413. 24009791 10.1371/journal.pntd.0002413 PMC3757062

[15] Glenn TC, Lance SL, McKee AM, Webster BL, Emery AM, Zerlotini A, et al. Significant variance in genetic diversity among populations of *Schistosoma haematobium* detected using microsatellite DNA loci from a genome-wide database. Parasit Vectors. 2013;6:300. 24499537 10.1186/1756-3305-6-300 PMC3874762

[16] Huyse T, Van den Broeck F, Hellemans B, Volckaert FAM, Polman K. Hybridisation between the two major African schistosome species of humans. Int J Parasitol. 2013;43:687–9. 23643461 10.1016/j.ijpara.2013.04.001

[17] Kane RA, Stothard JR, Rollinson D, Leclipteux T, Evraerts J, Standley CJ, et al. Detection and quantification of schistosome DNA in freshwater snails using either fluorescent probes in real-time PCR or oligochromatographic dipstick assays targeting the ribosomal intergenic spacer. Acta Trop. 2013;128:241–9. 22100540 10.1016/j.actatropica.2011.10.019

[18] Lopes JLS, Orcia D, Araujo APU, DeMarco R, Wallace BA. Folding factors and partners for the intrinsically disordered protein micro-exon gene 14 (MEG-14). Biophys J. 2013;104:2512–20. 23746524 10.1016/j.bpj.2013.03.063 PMC3672892

[19] Sealey KL, Kirk RS, Walker AJ, Rollinson D, Lawton SP. Adaptive radiation within the vaccine target tetraspanin-23 across nine *Schistosoma* species from Africa. Int J Parasitol. 2013;43:95–103. 23220042 10.1016/j.ijpara.2012.11.007

[20] Stothard JR, Sousa-Figueiredo JC, Navaratnam AM. Advocacy, policies and practicalities of preventive chemotherapy campaigns for African children with schistosomiasis. Expert Rev Anti Infect Ther. 2013;11:733–52. 23879611 10.1586/14787210.2013.811931

[21] Utzinger J, Brattig NW, Kristensen TK. Schistosomiasis research in Africa: how the CONTRAST alliance made it happen. Acta Trop. 2013;128:182–95. 23973364 10.1016/j.actatropica.2013.08.011

[22] Knopp S, Person B, Ame SM, Mohammed KA, Ali SM, Khamis IS, et al. Elimination of schistosomiasis transmission in Zanzibar: baseline findings before the onset of a randomized intervention trial. PLoS Negl Trop Dis. 2013;7: e2474. 24147165 10.1371/journal.pntd.0002474 PMC3798599

[23] Moser W, Greter H, Schindler C, Allan F, Ngandolo BNR, Moto DD, et al. The spatial and seasonal distribution of *Bulinus truncatus*, *Bulinus forskalii* and *Biomphalaria pfeifferi*, the intermediate host snails of schistosomiasis, in N’Djamena, Chad. Geospat Health. 2014;9:109. 25545929 10.4081/gh.2014.9

[24] Stothard JR, Stanton MC, Bustinduy AL, Sousa-Figueiredo JC, Van Dam GJ, Betson M, et al. Diagnostics for schistosomiasis in Africa and Arabia: a review of present options in control and future needs for elimination. Parasitology. 2014;141:1947–61. 25158604 10.1017/S0031182014001152

[25] Rosser A, Rollinson D, Forrest M, Webster B. Isothermal recombinase polymerase amplification (RPA) of *Schistosoma haematobium* DNA and oligochromatographic lateral flow detection. Parasit Vectors. 2015;8:446. 26338510 10.1186/s13071-015-1055-3 PMC4559068

[26] Van den Broeck F, Maes GE, Larmuseau MH, Rollinson D, Sy I, Faye D, et al. Reconstructing colonization dynamics of the human parasite *Schistosoma mansoni* following anthropogenic environmental changes in northwest Senegal. PLoS Negl Trop Dis. 2015;9: e0003998. 26275049 10.1371/journal.pntd.0003998 PMC4537236

[27] Webster BL, Rabone M, Pennance T, Emery AM, Allan F, Gouvras A, et al. Development of novel multiplex microsatellite polymerase chain reactions to enable high-throughput population genetic studies of *Schistosoma haematobium*. Parasit Vectors. 2015;8:1–5. 26329827 10.1186/s13071-015-1044-6 PMC4557312

[28] Crellen T, Allan F, David S, Durrant C, Huckvale T, Holroyd N, et al. Whole genome resequencing of the human parasite *Schistosoma mansoni* reveals population history and effects of selection. Sci Rep. 2016;6:20954. 26879532 10.1038/srep20954 PMC4754680

[29] Easton AVA. Assessing the impact of mass deworming: changes in soil-transmitted helminth burden and characteristics, co-infections and the gut microbiome [PhD]. Imperial College London; 2016. http://spiral.imperial.ac.uk/handle/10044/1/68284. Accesesd 19 Dec 2024.

[30] Knopp S, Person B, Ame SM, Ali SM, Muhsin J, Juma S, et al. Praziquantel coverage in schools and communities targeted for the elimination of urogenital schistosomiasis in Zanzibar: a cross-sectional survey. Parasit Vectors. 2016;9:5. 26727915 10.1186/s13071-015-1244-0 PMC4700672

[31] Léger E, Garba A, Hamidou AA, Webster BL, Pennance T, Rollinson D, et al. Introgressed animal schistosomes *Schistosoma curassoni* and *S. bovis* naturally infecting humans. Emerg Infect Dis. 2016;22:2212–4. 27869609 10.3201/eid2212.160644 PMC5189150

[32] Nussbeck SY, Rabone M, Benson EE, Droege G, Mackenzie-Dodds J, Lawlor RT. “Life in data”—outcome of a multi-disciplinary, interactive biobanking conference session on sample data. Biopreserv Biobank. 2016;14:56–64. 26808538 10.1089/bio.2015.0061 PMC4761830

[33] Pennance T, Person B, Muhsin MA, Khamis AN, Muhsin J, Khamis IS, et al. Urogenital schistosomiasis transmission on Unguja Island, Zanzibar: characterisation of persistent hot-spots. Parasit Vectors. 2016;9:646. 27986092 10.1186/s13071-016-1847-0 PMC5162088

[34] Abbasi I, Webster BL, King CH, Rollinson D, Hamburger J. The substructure of three repetitive DNA regions of *Schistosoma haematobium* group species as a potential marker for species recognition and interbreeding detection. Parasit Vectors. 2017;10:364. 28764739 10.1186/s13071-017-2281-7 PMC5540583

[35] Allan F, Sousa-Figueiredo JC, Emery AM, Paulo R, Mirante C, Sebastião A, et al. Mapping freshwater snails in north-western Angola: distribution, identity and molecular diversity of medically important taxa. Parasit Vectors. 2017;10:460. 29017583 10.1186/s13071-017-2395-y PMC5634851

[36] Crellen T. Population genomics and epidemiology of *Schistosoma mansoni* [PhD]. Imperial College London; 2017. https://core.ac.uk/download/pdf/189833823.pdf. Accessed 19 Dec 2024.

[37] Gouvras AN, Allan F, Kinung’hi S, Rabone M, Emery A, Angelo T, et al. Longitudinal survey on the distribution of *Biomphalaria sudanica* and *B. choanomophala* in Mwanza region, on the shores of Lake Victoria, Tanzania: implications for schistosomiasis transmission and control. Parasit Vectors. 2017;10:316. 28659165 10.1186/s13071-017-2252-z PMC5490224

[38] World Health Organization. Field use of molluscicides in schistosomiasis control programmes: an operational manual for programme managers. 1st ed. Geneva: World Health Organization; 2017.

[39] Abe EM, Guo Y-H, Shen H, Mutsaka-Makuvaza MJ, Habib MR, Xue J-B, et al. Phylogeography of *Bulinus truncatus* (Audouin, 1827)(Gastropoda: Planorbidae) in selected African countries. Trop Med Infect Dis. 2018;3:127. 30572694 10.3390/tropicalmed3040127 PMC6306716

[40] Anderson TJC, LoVerde PT, Le Clec’h W, Chevalier FD. Genetic crosses and linkage mapping in schistosome parasites. Trends Parasitol. 2018;34:982–96. 30150002 10.1016/j.pt.2018.08.001 PMC6382074

[41] Boon NA, Van den Broeck F, Faye D, Volckaert FA, Mboup S, Polman K, et al. Barcoding hybrids: heterogeneous distribution of *Schistosoma haematobium* times *Schistosoma bovis* hybrids across the Senegal River Basin. Parasitology. 2018;145:634–45. 29667570 10.1017/S0031182018000525

[42] Booth M, Clements A. Neglected tropical disease control—the case for adaptive, location-specific solutions. Trends Parasitol. 2018;34:272–82. 29500033 10.1016/j.pt.2018.02.001

[43] Lawton SP, Allan F, Hayes PM, Smit NJ. DNA barcoding of the medically important freshwater snail *Physa acuta* reveals multiple invasion events into Africa. Acta Trop. 2018;188:86–92. 30165073 10.1016/j.actatropica.2018.08.027

[44] Le Clec’h W, Chevalier FD, McDew-White M, Allan F, Webster BL, Gouvras AN, et al. Whole genome amplification and exome sequencing of archived schistosome miracidia. Parasitology. 2018;145:1739–47. 29806576 10.1017/S0031182018000811 PMC6193844

[45] Pennance T, Ame SM, Amour AK, Suleiman KR, Allan F, Rollinson D, et al. Occurrence of Schistosoma bovis on Pemba Island, Zanzibar: implications for urogenital schistosomiasis transmission monitoring. Parasitology. 2018;145:1727–31. 30086805 10.1017/S0031182018001154 PMC7116046

[46] Poulton K, Webster B. Development of a lateral flow recombinase polymerase assay for the diagnosis of *Schistosoma mansoni* infections. Anal Biochem. 2018;546:65–71. 29425749 10.1016/j.ab.2018.01.031

[47] Sene-Wade M, Marchand B, Rollinson D, Webster BL. Urogenital schistosomiasis and hybridization between *Schistosoma haematobium* and *Schistosoma bovis* in adults living in Richard-Toll, Senegal. Parasitology. 2018;145:1723–6. 30185248 10.1017/S0031182018001415

[48] Tian-Bi Y-NT, Ouattara M, Knopp S, Coulibaly JT, Hürlimann E, Webster B, et al. Interrupting seasonal transmission of *Schistosoma haematobium* and control of soil-transmitted helminthiasis in northern and central Côte d’Ivoire: a SCORE study protocol. BMC Public Health. 2018;18:186. 29378542 10.1186/s12889-018-5044-2 PMC5789673

[49] Boon NAM, Mbow M, Paredis L, Moris P, Sy I, Maes T, et al. No barrier breakdown between human and cattle schistosome species in the Senegal River Basin in the face of hybridisation. Int J Parasitol. 2019;49:1039–48. 31734338 10.1016/j.ijpara.2019.08.004

[50] Catalano S, Symeou A, Marsh KJ, Borlase A, Léger E, Fall CB, et al. Mini-FLOTAC as an alternative, non-invasive diagnostic tool for *Schistosoma mansoni* and other trematode infections in wildlife reservoirs. Parasit Vectors. 2019;12:1–9. 31522684 10.1186/s13071-019-3613-6 PMC6745783

[51] Chevalier FD, Clec’h WL, McDew-White M, Menon V, Guzman MA, Holloway SP, et al. Oxamniquine resistance alleles are widespread in Old World *Schistosoma mansoni* and predate drug deployment. PLOS Pathog. 2019;15: e1007881. 31652296 10.1371/journal.ppat.1007881 PMC6834289

[52] Doyle SR, Sankaranarayanan G, Allan F, Berger D, Jimenez Castro PD, Collins JB, et al. Evaluation of DNA extraction methods on individual helminth egg and larval stages for whole-genome sequencing. Front Genet. 2019;10:826. 31616465 10.3389/fgene.2019.00826 PMC6764475

[53] Harmon A, Littlewood DTJ, Wood CL. Parasites lost: using natural history collections to track disease change across deep time. Front Ecol Environ. 2019;17:157–66.

[54] Coghlan A, Tyagi R, Cotton JA, Holroyd N, Rosa BA, Tsai IJ, et al. Comparative genomics of the major parasitic worms. Nat Genet. 2019;51:163–74. 30397333 10.1038/s41588-018-0262-1 PMC6349046

[55] Oey H, Zakrzewski M, Gravermann K, Young ND, Korhonen PK, Gobert GN, et al. Whole-genome sequence of the bovine blood fluke *Schistosoma bovis* supports interspecific hybridization with *S. haematobium*. PLOS Pathog. 2019;15: e1007513. 30673782 10.1371/journal.ppat.1007513 PMC6361461

[56] Papaiakovou M, Gasser RB, Littlewood DTJ. Quantitative PCR-based diagnosis of soil-transmitted helminth infections: faecal or fickle? Trends Parasitol. 2019;35:491–500. 31126720 10.1016/j.pt.2019.04.006

[57] Platt RN, McDew-White M, Le Clec’h W, Chevalier FD, Allan F, Emery AM, et al. Ancient hybridization and adaptive introgression of an invadolysin gene in schistosome parasites. Mol Biol Evol. 2019;36:2127–42. 31251352 10.1093/molbev/msz154 PMC6759076

[58] Rabone M, Wiethase JH, Allan F, Gouvras AN, Pennance T, Hamidou AA, et al. Freshwater snails of biomedical importance in the Niger River Valley: evidence of temporal and spatial patterns in abundance, distribution and infection with *Schistosoma* spp. Parasit Vectors. 2019;12:498. 31640811 10.1186/s13071-019-3745-8 PMC6805334

[59] Rostron P, Pennance T, Bakar F, Rollinson D, Knopp S, Allan F, et al. Development of a recombinase polymerase amplification (RPA) fluorescence assay for the detection of *Schistosoma haematobium*. Parasit Vectors. 2019;12:514. 31685024 10.1186/s13071-019-3755-6 PMC6827214

[60] Tian-Bi Y-NT, Webster B, Konan CK, Allan F, Diakité NR, Ouattara M, et al. Molecular characterization and distribution of *Schistosoma* cercariae collected from naturally infected bulinid snails in northern and central Côte d’Ivoire. Parasit Vectors. 2019;12:117. 30890180 10.1186/s13071-019-3381-3 PMC6423847

[61] Wood CL, Sokolow SH, Jones IJ, Chamberlin AJ, Lafferty KD, Kuris AM, et al. Precision mapping of snail habitat provides a powerful indicator of human schistosomiasis transmission. Proc Natl Acad Sci. 2019;116:23182–91. 31659025 10.1073/pnas.1903698116 PMC6859407

[62] Allan F, Ame SM, Tian-Bi Y-NT, Hofkin BV, Webster BL, Diakité NR, et al. Snail-Related contributions from the schistosomiasis consortium for operational research and evaluation program including xenomonitoring, focal mollusciciding, biological control, and modeling. Am J Trop Med Hyg. 2020;103:66–79. 32400353 10.4269/ajtmh.19-0831 PMC7351297

[63] Archer J, Barksby R, Pennance T, Rostron P, Bakar F, Knopp S, et al. Analytical and clinical assessment of a portable, isothermal recombinase polymerase amplification (RPA) assay for the molecular diagnosis of urogenital schistosomiasis. Molecules. 2020;25:4175. 32933094 10.3390/molecules25184175 PMC7570534

[64] Catalano S, Léger E, Fall CB, Borlase A, Diop SD, Berger D, et al. Multihost Transmission of *Schistosoma mansoni* in Senegal, 2015–2018. Emerg Infect Dis. 2020;26:1234–42. 32441625 10.3201/eid2606.200107 PMC7258455

[65] Colley DG, Fleming FM, Matendechero SH, Knopp S, Rollinson D, Utzinger J, et al. Contributions of the Schistosomiasis Consortium for Operational Research and Evaluation (SCORE) to schistosomiasis control and elimination: key findings and messages for future goals, thresholds, and operational research. Am J Trop Med Hyg. 2020;103:125–34. 32400345 10.4269/ajtmh.19-0787 PMC7351304

[66] Keller D, Rothen J, Dangy J-P, Saner C, Daubenberger C, Allan F, et al. Performance of a real-time PCR approach for diagnosing *Schistosoma haematobium* infections of different intensity in urine samples from Zanzibar. Infect Dis Poverty. 2020;9:128. 32887642 10.1186/s40249-020-00726-y PMC7487541

[67] Léger E, Borlase A, Fall CB, Diouf ND, Diop SD, Yasenev L, et al. Prevalence and distribution of schistosomiasis in human, livestock, and snail populations in northern Senegal: a One Health epidemiological study of a multi-host system. Lancet Planet Health. 2020;4:e330–42. 32800151 10.1016/S2542-5196(20)30129-7 PMC7443702

[68] Pennance T. Genetic diversity and evolution within the genus Bulinus and species-level interactions with the transmission of *Schistosoma haematobium* group parasites [PhD]. Cardiff University; 2020. https://orca.cardiff.ac.uk/id/eprint/135638/. Accessed 1 Sep 2024.

[69] Pennance T, Allan F, Emery A, Rabone M, Cable J, Garba AD, et al. Interactions between *Schistosoma haematobium* group species and their *Bulinus* spp. intermediate hosts along the Niger River Valley. Parasit Vectors. 2020;13:268. 32448268 10.1186/s13071-020-04136-9 PMC7247258

[70] Pennance T, Archer J, Lugli EB, Rostron P, Llanwarne F, Ali SM, et al. Development of a molecular snail xenomonitoring assay to detect *Schistosoma haematobium* and *Schistosoma bovis* infections in their *Bulinus* snail hosts. Molecules. 2020;25:4011. 32887445 10.3390/molecules25174011 PMC7116084

[71] Webster JP, Neves MI, Webster BL, Pennance T, Rabone M, Gouvras AN, et al. Parasite population genetic contributions to the Schistosomiasis Consortium for Operational Research and Evaluation within Sub-Saharan Africa. Am J Trop Med Hyg. 2020;103:80–91. 32400355 10.4269/ajtmh.19-0827 PMC7351308

[72] Berger D. Comparative and population genomic analyses of the parasitic blood fluke [PhD]. University of Cambridge; 2021. 10.17863/CAM.86667.

[73] Berger DJ, Crellen T, Lamberton PHL, Allan F, Tracey A, Noonan JD, et al. Whole-genome sequencing of *Schistosoma mansoni* reveals extensive diversity with limited selection despite mass drug administration. Nat Commun. 2021;12:4776. 34362894 10.1038/s41467-021-24958-0 PMC8346512

[74] Halili S, Grant JR, Pilotte N, Gordon CA, Williams SA. Development of a novel real-time polymerase chain reaction assay for the sensitive detection of *Schistosoma japonicum* in human stool. PLoS Negl Trop Dis. 2021;15: e0009877. 34695134 10.1371/journal.pntd.0009877 PMC8568117

[75] Le Clec’h W, Chevalier FD, Mattos ACA, Strickland A, Diaz R, McDew-White M, et al. Genetic analysis of praziquantel response in schistosome parasites implicates a transient receptor potential channel. Sci Transl Med. 2021;13: eabj9114. 34936381 10.1126/scitranslmed.abj9114 PMC9491494

[76] Rey O, Toulza E, Chaparro C, Allienne J-F, Kincaid-Smith J, Mathieu-Begné E, et al. Diverging patterns of introgression from *Schistosoma bovis* across *S. haematobium* African lineages. PLOS Pathog. 2021;17: e1009313. 33544762 10.1371/journal.ppat.1009313 PMC7891765

[77] Tallam K, Liu ZY-C, Chamberlin AJ, Jones IJ, Shome P, Riveau G, et al. Identification of snails and *Schistosoma* of medical importance via convolutional neural networks: a proof-of-concept application for human schistosomiasis. Front Public Health. 2021;9:642895. 34336754 10.3389/fpubh.2021.642895 PMC8319642

[78] Thompson CW, Phelps KL, Allard MW, Cook JA, Dunnum JL, Ferguson AW, et al. Preserve a voucher specimen! The critical need for integrating natural history collections in infectious disease studies. MBio. 2021. 10.1128/mbio.02698-20. 33436435 10.1128/mBio.02698-20 PMC7844540

[79] Berger DJ, Léger E, Sankaranarayanan G, Sène M, Diouf ND, Rabone M, et al. Genomic evidence of contemporary hybridization between *Schistosoma* species. PLOS Pathog. 2022;18: e1010706. 35939508 10.1371/journal.ppat.1010706 PMC9387932

[80] Landeryou T, Rabone M, Allan F, Maddren R, Rollinson D, Webster BL, et al. Genome-wide insights into adaptive hybridisation across the *Schistosoma haematobium* group in West and Central Africa. PLoS Negl Trop Dis. 2022;16: e0010088. 35100291 10.1371/journal.pntd.0010088 PMC8803156

[81] Mesquita SG, Lugli EB, Matera G, Fonseca CT, Caldeira RL, Webster B. Development of real-time and lateral flow recombinase polymerase amplification assays for rapid detection of *Schistosoma mansoni*. Front Microbiol. 2022;13:1043596. 36466644 10.3389/fmicb.2022.1043596 PMC9716991

[82] Miswan N, Singham GV, Othman N. Advantages and limitations of microscopy and molecular detections for diagnosis of soil-transmitted helminths: an overview. Helminthologia. 2022;59:321–40. 36875683 10.2478/helm-2022-0034 PMC9979072

[83] Moser W, Batil AA, Ott R, Abderamane M, Clements R, Wampfler R, et al. High prevalence of urinary schistosomiasis in a desert population: results from an exploratory study around the Ounianga lakes in Chad. Infect Dis Poverty. 2022;11:5. 34991728 10.1186/s40249-021-00930-4 PMC8740043

[84] Pennance T, Neves MI, Webster BL, Gower CM, Knopp S, Khamis IS, et al. Potential drivers for schistosomiasis persistence: population genetic analyses from a cluster-randomized urogenital schistosomiasis elimination trial across the Zanzibar islands. PLoS Negl Trop Dis. 2022;16: e0010419. 36215334 10.1371/journal.pntd.0010419 PMC9584424

[85] Stroehlein AJ, Korhonen PK, Vern Lee V, Ralph SA, Mentink-Kane M, You H, et al. Chromosome-level genome of *Schistosoma haematobium* underpins genome-wide explorations of molecular variation. PLoS Pathog. 2022;18: e1010288. 35167626 10.1371/journal.ppat.1010288 PMC8846543

[86] Webb AJ, Allan F, Kelwick RJR, Beshah FZ, Kinung’hi SM, Templeton MR, et al. Specific nucleic acid ligation for the detection of schistosomes: SNAILS. PLoS Negl Trop Dis. 2022;16: e0010632. 35881651 10.1371/journal.pntd.0010632 PMC9355235

[87] Cherkaoui D, Mesquita SG, Huang D, Lugli EB, Webster BL, McKendry RA. CRISPR-assisted test for *Schistosoma haematobium*. Sci Rep. 2023;13:4990. 36973334 10.1038/s41598-023-31238-y PMC10042105

[88] Senghor B, Webster B, Pennance T, Sène M, Doucouré S, Sow D, et al. Molecular characterization of schistosome cercariae and their *Bulinus* snail hosts from Niakhar, a seasonal transmission focus in central Senegal. Curr Res Parasitol Vector-Borne Dis. 2023;3:100114. 36824299 10.1016/j.crpvbd.2023.100114 PMC9941053

[89] Trippler L, Knopp S, Welsche S, Webster BL, Stothard JR, Blair L, et al. The long road to schistosomiasis elimination in Zanzibar: a systematic review covering 100 years of research, interventions and control milestones. Adv Parasitol. 2023;122:71–191. 37657854 10.1016/bs.apar.2023.06.001

[90] Ajakaye OG, Enabulele EE, Balogun JB, Oyeyemi OT, Grigg ME. Extant interspecific hybridization among trematodes within the *Schistosoma haematobium* species complex in Nigeria. PLoS Negl Trop Dis. 2024;18: e0011472. 38620029 10.1371/journal.pntd.0011472 PMC11045100

[91] Andrus PS, Joof E, Wade CM. Differentiation of *Bulinus senegalensis* and *Bulinus forskalii* Snails in West Africa using morphometric analysis. Acta Parasitol. 2024;69:1016–26. 38502474 10.1007/s11686-024-00830-1 PMC11001693

[92] Archer J, Yeo SM, Gadd G, Pennance T, Cunningham LJ, Juhàsz A, et al. Development, validation, and pilot application of a high throughput molecular xenomonitoring assay to detect *Schistosoma mansoni* and other trematode species within *Biomphalaria* freshwater snail hosts. Curr Res Parasitol Vector-Borne Dis. 2024;5:100174. 38618156 10.1016/j.crpvbd.2024.100174 PMC11010794

[93] Berger DJ, Park S-K, Crellen T, Vianney TJ, Kabatereine NB, Cotton JA, et al. Extensive transmission and variation in a functional receptor for praziquantel resistance in endemic *Schistosoma mansoni*. bioRxiv. 2024. 10.1101/2024.08.29.610291v2.39763900

[94] Donnelly O, Mesquita S, Archer J, Ali SM, Bartonicek Z, Lugli EB, et al. Refining the *Schistosoma haematobium* recombinase polymerase amplification (Sh-RPA) assay: moving towards point-of-care use in endemic settings. Parasit Vectors. 2024;17:321. 39068490 10.1186/s13071-024-06380-9 PMC11283713

[95] Platt RN, Enabulele EE, Adeyemi E, Agbugui MO, Ajakaye OG, Amaechi EC, et al. Genomic data reveal a north-south split and introgression history of blood fluke (*Schistosoma haematobium*) populations from across Africa. bioRxiv. 2024. 10.1101/2024.08.06.606828v1.39484487

[96] Stelbrink B, Kehlmaier C, Clewing C, Wilke T, Albrecht C. A genetic snapshot before extinction: Museomics reveals the phylogenetic position of a critically endangered freshwater gastropod. Aquat Conserv Mar Freshw Ecosyst. 2024;34: e4226.

[97] Gower CM, Shrivastava J, Lamberton PHL, Rollinson D, Webster BL, Emery A, et al. Development and application of an ethically and epidemiologically advantageous assay for the multi-locus microsatellite analysis of *Schistosoma mansoni*. Parasitology. 2007;134:523–36. 17096873 10.1017/S0031182006001685 PMC2613677

[98] Agola EL, Mwangi IN, Maina GM, Kinuthia JM, Mutuku MW. Transmission sites for *Schistosoma haematobium* and *Schistosoma bovis* identified in localities within the Athi River basin of Kenya using a PCR-RFLP assay. Heliyon. 2021;7: e06114. 33644442 10.1016/j.heliyon.2021.e06114 PMC7889825

[99] Betson M, Sousa-Figueiredo JC, Kabatereine NB, Stothard JR. New insights into the molecular epidemiology and population genetics of *Schistosoma mansoni* in Ugandan pre-school children and mothers. PLoS Negl Trop Dis. 2013;7: e2561. 24349589 10.1371/journal.pntd.0002561 PMC3861247

[100] Van den Broeck F, Geldof S, Polman K, Volckaert FAM, Huyse T. Optimal sample storage and extraction procotols for reliable multilocus genotyping of the human parasite *Schistosoma mansoni*. Infect Genet Evol. 2011;11:1413–8. 21605705 10.1016/j.meegid.2011.05.006

[101] Xiao N, Remais JV, Brindley PJ, Qiu D-C, Carlton EJ, Li R-Z, et al. Approaches to genotyping individual miracidia of *Schistosoma japonicum*. Parasitol Res. 2013;112:3991–9. 24013341 10.1007/s00436-013-3587-9 PMC3834234

[102] Visser PS, Pitchford RJ. A simple apparatus for rapid recovery of helminth eggs from excreta with special reference to *Schistosoma mansoni*. South Afr Med J Suid-Afr Tydskr Vir Geneeskd. 1972;46:1344–6. 4674019

[103] Moore L, Leongamornlert D, Coorens THH, Sanders MA, Ellis P, Dentro SC, et al. The mutational landscape of normal human endometrial epithelium. Nature. 2020;580:640–6. 32350471 10.1038/s41586-020-2214-z

[104] Webster BL, Webster JP, Gouvras AN, Garba A, Lamine MS, Diaw OT, et al. DNA ‘barcoding’ of *Schistosoma mansoni* across sub-Saharan Africa supports substantial within locality diversity and geographical separation of genotypes. Acta Trop. 2013;128:250–60. 22935316 10.1016/j.actatropica.2012.08.009

[105] Tchuenté LAT, Southgate VR, Njiokou F, Njiné T, Kouemeni LE, Jourdane J. The evolution of schistosomiasis at Loum, Cameroon: replacement of *Schistosoma intercalatum* by *S. haematobium* through introgressive hybridization. Trans R Soc Trop Med Hyg. 1997;91:664–5. 9509173 10.1016/s0035-9203(97)90513-7

[106] Léger E, Webster JP. Hybridizations within the Genus *Schistosoma*: implications for evolution, epidemiology and control. Parasitology. 2017;144:65–80. 27572906 10.1017/S0031182016001190

[107] Webster BL, Diaw OT, Seye MM, Webster JP, Rollinson D. Introgressive hybridization of *Schistosoma haematobium* group species in Senegal: species barrier break down between ruminant and human Schistosomes. PLoS Negl Trop Dis. 2013;7: e2110. 23593513 10.1371/journal.pntd.0002110 PMC3617179

[108] Kane RA, Bartley J, Stothard JR, Vercruysse J, Rollinson D, Southgate VR. Application of single strand conformational polymorphism (SSCP) analysis with fluorescent primers for differentiation of *Schistosoma haematobium* group species. Trans R Soc Trop Med Hyg. 2002;96:S235–41. 12055845 10.1016/s0035-9203(02)90082-9

[109] Summers S, Bhattacharyya T, Allan F, Stothard JR, Edielu A, Webster BL, et al. A review of the genetic determinants of praziquantel resistance in *Schistosoma mansoni*: is praziquantel and intestinal schistosomiasis a perfect match? Front Trop Dis. 2022;3:933097.

[110] Park S-K, Friedrich L, Yahya NA, Rohr CM, Chulkov EG, Maillard D, et al. Mechanism of praziquantel action at a parasitic flatworm ion channel. Sci Transl Med. 2021;13: eabj5832. 34936384 10.1126/scitranslmed.abj5832 PMC8855674

[111] Grove DI. A history of human helminthology. Wallingford: C.A.B. International; 1990.

[112] Conroy MC, Lacey B, Bešević J, Omiyale W, Feng Q, Effingham M, et al. UK Biobank: a globally important resource for cancer research. Br J Cancer. 2023;128:519–27. 36402876 10.1038/s41416-022-02053-5 PMC9938115

[113] Angeles NAC, Catap ES. Challenges on the development of biodiversity biobanks: the living archives of biodiversity. Biopreserv Biobank. 2023;21:5–13. 35133889 10.1089/bio.2021.0127

[114] Xu A, Baysari MT, Stocker SL, Leow LJ, Day RO, Carland JE. Researchers’ views on, and experiences with, the requirement to obtain informed consent in research involving human participants: a qualitative study. BMC Med Ethics. 2020;21:93. 33008387 10.1186/s12910-020-00538-7 PMC7531157

[115] Tegegne MD, Melaku MS, Shimie AW, Hunegnaw DD, Legese MG, Ejigu TA, et al. Health professionals’ knowledge and attitude towards patient confidentiality and associated factors in a resource-limited setting: a cross-sectional study. BMC Med Ethics. 2022;23:26. 35287659 10.1186/s12910-022-00765-0 PMC8922732

[116] Doucet M, Yuille M, Georghiou L, Dagher G. Biobank sustainability: current status and future prospects. J Biorepos Sci Appl Med. 2017;5:1–7.

[117] Schistosome Snail Resource (SSR). https://www.nhm.ac.uk/our-science/research/projects/schistosome-snail-resource.html. Accessed 17 Dec 2024.

[118] Rabone M, Horton T, Humphries F, Lyal CH, Muraki Gottlieb H, Scholz AH, Vanagt T, Jaspars M. BBNJ Agreement: considerations for scientists and commercial end users of MGR at research, development and commercialization stages. In: Humphries F, editor. Decoding marine genetic resource governance under the BBNJ Agreement. Cham: Springer Nature Switzerland; 2025. p. 283–315. 10.1007/978-3-031-72100-7_14.

[119] Prathapan KD, Pethiyagoda R, Bawa KS, Raven PH, Rajan PD, 172 Co-signatories from 35 Countries. When the cure kills—CBD limits biodiversity research. Science. 2018;360:1405–6. 29954970 10.1126/science.aat9844

[120] Laird S, Wynberg R, Rourke M, Humphries F, Muller MR, Lawson C. Rethink the expansion of access and benefit sharing. Science. 2020;367:1200–2. 32165574 10.1126/science.aba9609

[121] Scholz AH, Freitag J, Lyal CHC, Sara R, Cepeda ML, Cancio I, et al. Multilateral benefit-sharing from digital sequence information will support both science and biodiversity conservation. Nat Commun. 2022;13:1086. 35197464 10.1038/s41467-022-28594-0 PMC8866420

[122] Mc Cartney AM, Head MA, Tsosie KS, Sterner B, Glass JR, Paez S, et al. Indigenous peoples and local communities as partners in the sequencing of global eukaryotic biodiversity. Npj Biodivers. 2023;2:1–12. 38693997 10.1038/s44185-023-00013-7 PMC11062294

[123] Hogg CJ, Belov K. Reply to DeWoody et al.: inequitable access to affordable sequencing limits the benefits from population genomic insights. Proc Natl Acad Sci USA. 2022;119: e2211129119. 36161930 10.1073/pnas.2211129119 PMC9546551

[124] Fatumo S, Yakubu A, Oyedele O, Popoola J, Attipoe DA, Eze-Echesi G, et al. Promoting the genomic revolution in Africa through the Nigerian 100K Genome Project. Nat Genet. 2022;54:531–6. 35534563 10.1038/s41588-022-01071-6

