# Supplementary material for: The research contribution of the Schistosomiasis Collection at the Natural History Museum (SCAN): highlights, challenges and future directions
Source: Infect Dis Poverty. 2025 Apr 18;14:29. doi: 10.1186/s40249-025-01302-y (PMC12007343; doi:10.1186/s40249-025-01302-y)
Supplement: Supplementary file 2 — Supplementary Material 2: Supplementary figure 1. Pitchford funnel dimensions. [file 40249_2025_1302_MOESM2_ESM.pdf]

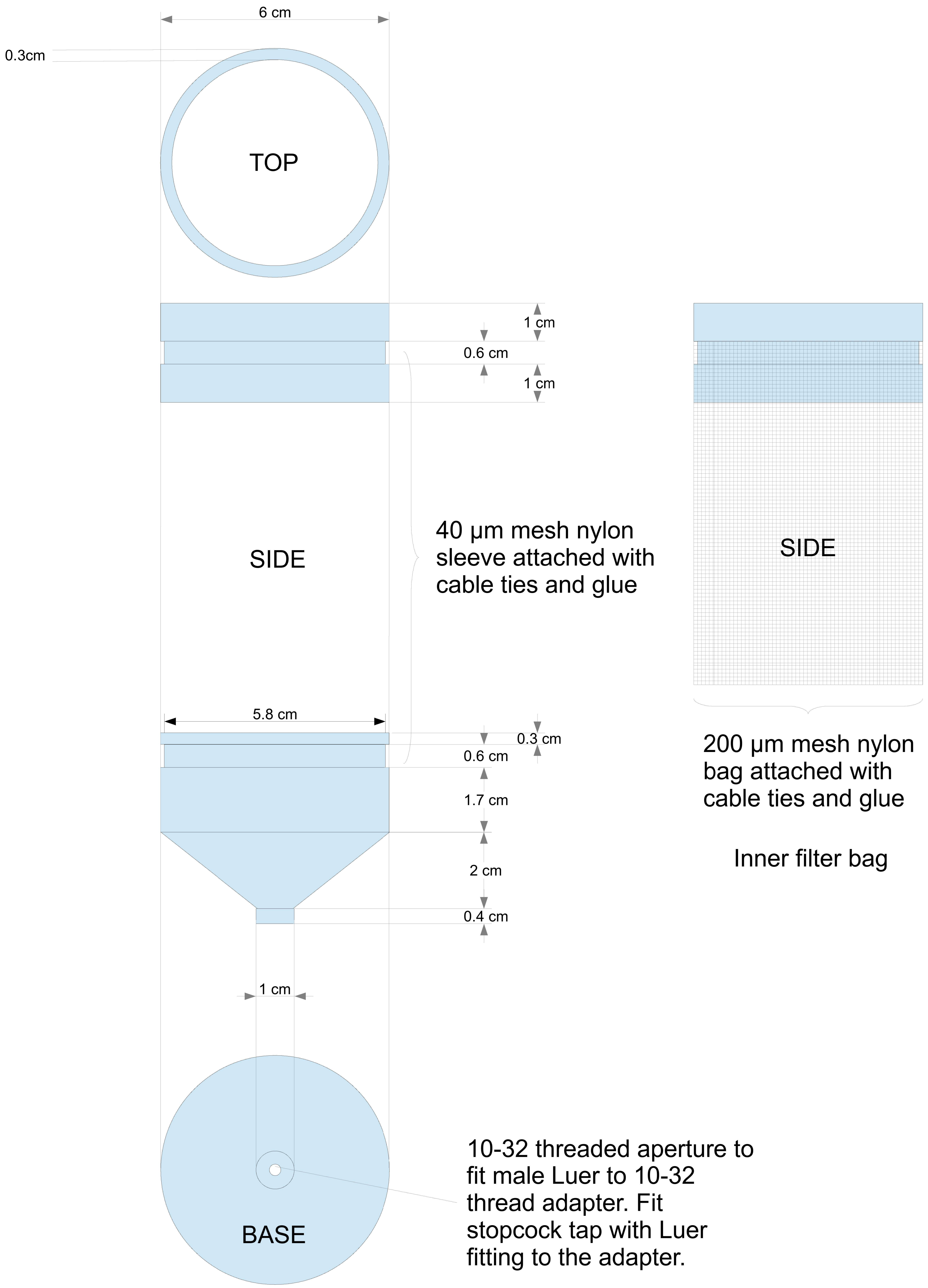

Outer funnel

Pitchford Funnel

Aidan Emery, 2024
